# Go with the Flow—Early Assessment of Measurable Residual Disease in Children with Acute Lymphoblastic Leukemia Treated According to ALL IC-BFM2009

**DOI:** 10.3390/cancers14215359

**Published:** 2022-10-30

**Authors:** Katarzyna Pawinska-Wasikowska, Karolina Bukowska-Strakova, Marta Surman, Monika Rygielska, Beata Sadowska, Teofila Ksiazek, Tomasz Klekawka, Aleksandra Wieczorek, Szymon Skoczen, Walentyna Balwierz

**Affiliations:** 1 Department of Pediatric Oncology and Hematology, Institute of Pediatrics, Jagiellonian University Medical College, 30-663 Krakow, Poland; 2 Department of Pediatric Oncology and Hematology, University Children’s Hospital, 30-663 Krakow, Poland; 3 Department of Clinical Immunology and Transplantation, Faculty of Medicine, Institute of Pediatrics, Jagiellonian University Medical College, 30-663 Krakow, Poland; 4 Hematology Laboratory, Department of Pediatric Oncology and Hematology, University Children’s Hospital, 30-663 Krakow, Poland; 5 Department of Pediatric Oncology and Hematology, Cytogenetics and Molecular Genetics Laboratory, University Children’s Hospital, 30-663 Krakow, Poland; 6 Department of Medical Genetics, Faculty of Medicine, Jagiellonian University Medical College, 30-663 Krakow, Poland

**Keywords:** acute lymphoblastic leukemia, minimal or measurable residual disease, blast cell clearance, flow cytometry, children

## Abstract

**Simple Summary:**

Monitoring of residual disease is a very important aspect of modern treatment approaches in many types of cancer. In acute leukemias in both children and adults, molecular and cytometric methods are used to assess the burden of leukemia at different points during therapy. Residual disease measured at the end of induction was shown to be the strongest predictor of outcome. Analyzing the outcomes of children with acute lymphoblastic leukemia (ALL), we aimed to establish the most informative cut-off and time point of assessment. Applying only the measurement of residual disease by flow cytometry along with genotypic findings, we managed to identify patients with a poor prognosis. Although new precise, molecular techniques as the next generation sequencing strategy are approaching daily clinical practice, flow cytometry is still a reliable, standardized method of residual disease detection. We may say ‘go with the flow’; thus, the assessment of residual disease by multiparametric flow cytometry is a proper method for the management of ALL patients according to risk-adapted therapies.

**Abstract:**

Measurable residual disease (MRD) is a well-known tool for the evaluation of the early response to treatment in patients with acute lymphoblastic leukemia (ALL). In respect to predicting the relapse the most informative cut-off and time point of MRD measurement during therapy were evaluated in our study. Between 1 January 2013 and 31 December 2019, multiparametric flow cytometry (MFC) MRD was measured in the bone marrow of 140 children with ALL treated according to the ALL IC-BFM2009 protocol. The MRD cut-off of 0.1% and day 33, end of induction, were the most discriminatory for all patients. Patients with negative MRD on day 15 and 33 had a higher 5-year overall survival—OS (100%) and a higher relapse-free survival—RFS rate (97.6%) than those with positive levels of MRD (≥0.01%) at both time points (77.8% and 55.6%, *p* = 0.002 and 0.001, respectively). Most patients with residual disease below 0.1% on day 15 exhibit hyperdiploidy or ETV6-RUNX1 in ALL cells. Measurement of MRD at early time points can be used with simplified genetic analysis to better identify low and high-risk patients, allowing personalized therapies and further improvement in outcomes in pediatric ALL.

## 1. Introduction

The presence of leukemic cells below the limit of detection by conventional cytomorphological methods is known as minimal or measurable residual disease (MRD). Persistent leukemic cells refractory to chemotherapy can be detected, identified, and measured by multiparameter flow cytometry (MFC), or molecular methods, such as quantitative polymerase chain reaction (PCR), or next-generation sequencing (NGS) techniques [1,2,3,4,5,6]. More sensitive methods than cytomorphology for the assessment of MRD have also redefined the state of remission in acute lymphoblastic leukemia (ALL), enabling the detection of leukemic cells in the bone marrow with less than 5% blasts and signs of hematopoietic recovery. However, patients with hematologic remission can still harbor residual leukemic cells, which could be the source of future relapse [1,2]. The presence of measurable residual leukemic cells after induction, as well as after consolidation chemotherapy, is a well-established prognostic factor that predicts a higher risk of relapse and shorter survival in leukemic patients regardless of their age or leukemia immunophenotype [7,8,9,10,11]. Since the early 1990s, different international study groups, such as Associazione Italiana Ematologia ed Oncologia Pediatrica (AEIOP), Berlin-Frankfurt-Munster (BFM), Dutch Childhood Oncology Group (DCOG), Children Oncology Group (COG), and Ma-Spore ALL, have shown that early response to therapy expressed by MRD load is strongly associated with the risk of relapse [12,13,14,15,16,17,18,19,20,21,22,23]. Therefore, the MRD analysis has become a crucial part of management strategy, helping to make the decision on whether to intensify or reduce therapy in children with acute leukemia according to personal risk assessment. MRD-directed risk-adapted therapies enabled tailoring optimal therapy for the individual patient minimizing toxicity and decreasing the risk of failure. Personalized therapy, based on the result of MRD measured during treatment, helps to find those among high-risk patients (e.g., with *IKZF1*^plus^), who have very good chances of cure with chemotherapy alone, if they achieve negative MRD at the end of induction. On the contrary, patients with positive MRD status, a proof of chemoresistance, could benefit from transplantation [24]. The sensitivity of the MRD analysis ranges from 0.1 to 0.001% (10^−1^ to 10^−5^) for MFC and 0.001 to 0.0001% (10^−5^ to 10^−6^) for molecular techniques. The NGS technique has become an accurate and sensitive method of MRD assessment, feasible to use in most of ALL cases, however its relatively high cost seems to be a serious obstacle for its wide applicability in daily practice. The detection of immunoglobulin and T cell receptor gene rearrangements by quantitative real-time PCR (RT-PCR) is considered the gold standard of care in the treatment of ALL [6,16,17,18,19,23,24,25,26]. However, most current therapeutic protocols use an integrated approach, applying both MFC and PCR techniques to assess MRD in ALL [12,13,14,26]. Broad applicability in ALL, along with availability in many laboratories, and rapid results in 24 h make MFC a widely used tool for MRD assessment. Therefore, MFC MRD monitoring is a well-established standard of care for early response assessment in ALL in the USA and Europe [12,13,14,15,16]. Due to its relatively low cost and improved standardization of the method, the MFC MRD strategy is also recommended in centers with limited resources [27,28]. In the AEIOP-BFM2000 study, measurement of MFC MRD on day 15 in bone marrow was the most powerful early predictor of relapse [13]. MRD at the end of induction (EOI) and/or consolidation (EOC) has been shown to be a significant prognostic tool in many international studies [7,11,12,13,14,15,16,29]. However, a single threshold for assigning patients to the MRD risk group does not reflect the response kinetics. There are still some patients who, regardless of high leukemia burden at the early time points of the assessment, present low or undetectable MRD at later measurement points. Early blast clearance, measured by MFC, allows for continuous assessment of leukemic burden in bone marrow, making the kinetics of its clearance also an informative indicator of outcome in childhood acute lymphoblastic leukemia [3,16,20]. In this study, we report the applicability of MFC MRD in the assessment of early blast clearance and its impact on outcomes of children with acute lymphoblastic leukemia treated according to the ALL IC-BFM2009 protocol, defining the most informative time point, cut-off, and clearance kinetics for this cohort of patients, as well as the wide applicability of the MFC method. The purpose of the study was also to determine the results of the ALL IC-BFM2009 study in our center.

## 2. Materials and Methods

### 2.1. Patients

Between January 2013 and December 2019, one hundred forty-seven consecutively recruited children, aged 1–18 years, with newly diagnosed ALL were treated in the Department of Pediatric Oncology and Hematology of the Krakow University Children’s Hospital in Poland according to ALL IC-BFM2009. Of these patients, 140 children were eligible for assessment (53 girls, 87 boys). Infants younger than 1 and ALL cases positive for BCR-ABL1 were not included in the study. The former were treated according to the Interfant-99 or-06 protocol, and the latter according to the EsPhALL protocol. Patients were eligible for final analysis based on the availability of an initial leukemia-associated immunophenotype (LAIP) and at least two bone marrow follow-up samples for the evaluation of MRD. The study protocol has been carried out in accordance with the code of ethics of the World Medical Association (Declaration of Helsinki) for experiments involving humans. Informed consent was obtained from parents/guardians.

### 2.2. Diagnosis and Assessment of MRD

Diagnosis of ALL was based on standard morphologic, cytochemical, immunophenotype, and genetic studies. Flow cytometry assessment was performed immediately after the bone marrow sample was obtained. For each patient, malignant cells were tested at diagnosis and LAIP was determined, which was used afterwards for MRD measurements during induction therapy in bone marrow samples. Appropriate volume of BM samples (1 × 10^6^ of leukocytes) was stained with a combination of 6 color (before 2015) or 10 color (after 2015) combination of monoclonal antibodies. Table 1 (panels A,B,C) contains panels of monoclonal antibodies used for MRD monitoring in our study. Example of the gating strategy is shown in Supplementary Materials (Figure S1,/panels A,B,C).

After staining, the samples were lysed and fixed with FACS Lysing Buffer. To identify nucleated cells, Syto41 was added just before acquisition. Cells were acquired using a FASCanto 10-color flow cytometer (Becton Dickinson) and analyzed using FACSDiva version 8.0.1 software. To obtain the appropriate sensitivity of the method (at least 10^−4^), at least 300,000 nucleated cells (NC) were acquired. Sample quality was assessed based on the percentage of erythroblasts. Samples containing less than 2% erythroblasts were classified as poor quality, were not used for evaluation, and bone marrow aspiration was repeated.

The minimal number of detectable blasts required to consider MRD positive was a group of 10 events (≥0.01%), and the MRD results were given as the percentage of blasts between total nucleated cells expressing CD45. MRD was related only to NC-positive cells with SYTO-41. Samples for MRD detection were obtained at several time points during induction therapy: day 0 (TP0), 15 (TP1), 33 (TP2), at the end of induction (EOI) and day 78 (TP3), at the end of consolidation (EOC). According to ALL IC-BFM 2009 protocol, the assessment of MFC MRD on day 15 has been applied to the assignment of risk groups and the selection of treatment (Table 2).

Cytogenetic techniques were used for the evaluation of every patient diagnosed with ALL. Fusion transcripts of t(12;21)/ETV6-RUNX1, t(1;19)/TCF3-PBX1, t(9;22)/BCR-ABL1, t(9;12)/PAX5-ETV6, and 11q23/KMT2A rearrangements were assessed using reverse transcription polymerase chain reaction and/or fluorescence in situ hybridization.

### 2.3. Stratification—Allocation to Treatment Groups

The patients were stratified according to the used protocol into three risk groups after induction therapy: standard risk (SR), intermediate risk (IR), high risk (HR). Stratification was based on age, white leukocyte count at diagnosis, response to therapy measured by blast count in peripheral blood after 7 days of corticotherapy, bone marrow blast count on days 15 and 33 of induction therapy, as well as MFC MRD on day 15. The cut-off point for MFC MRD was 0.1% of blasts in the bone marrow. MRD ≥ 10% on day 15 and hypodiploidy ≤ 44 chromosomes in leukemic cells upgraded patient to HR. SR patients should have had < 0.1% in bone marrow on day 15 by MFC MRD.

Prognostic factors used for stratification are presented in Table 2.

### 2.4. Treatment

The ALL IC-BFM2009 protocol used a therapy schedule based on the original BFM backbone. At first, all patients received one week of prednisone pre-phase, along with intrathecal methotrexate. The induction chemotherapy with corticosteroids, vincristine, daunorubicin, L-asparaginase, and intrathecal methotrexate was then introduced (Protocol I, phase 1). In standard-risk patients, two doses of daunorubicin were administered, instead of four. In consolidation therapy, cyclophosphamide, cytarabine, mercaptopurine were used (Protocol I, phase 2). SR and IR groups: Subsequently, patients in the standard and intermediate risk group received methotrexate and mercaptopurine. SR BCP-ALL patients received four courses of methotrexate 2 g/m^2^ (Protocol mM) and SR T-ALL patients, as well as all IR patients receiving high-dose methotrexate (5 g/m^2^) four times in Protocol M. Further treatment in SR and IR patients consisted of delayed intensification (Protocol II) with dexamethasone, vincristine, doxorubicin, L-asparaginase, cyclophosphamide, cytarabine, thioguanine, intrathecal methotrexate. Maintenance therapy, based on oral mercaptopurine and methotrexate, for a total duration of 2 years, was the last part of the therapy. CNS-directed therapy: In the event of CNS involvement, or in patients with TALL, triple intrathecal therapy (prednisolone/ARAC/MTX) was administered, followed by CNS radiotherapy. Intrathecal methotrexate was administered as a prophylactic measure.

HR group: After induction and consolidation therapy, HR patients received 6 HR blocks (HR-1, HR-2, HR-3, twice each) followed by Protocol II and maintenance therapy or 3 HR blocks followed by allogeneic hematopoietic stem cell transplantation (allo-HSCT). Corticosensitivity was defined as less than 1 × 10^9^/L in peripheral blood on day 8 and chemosensitivity by less than 5% of blasts in the bone marrow on day 15 and/or 33. Complete remission (CR) was evaluated in bone marrow on day 15 and/or 33, tested both morphologically and by flow cytometry.

### 2.5. Statistical Analysis

Associations between MRD, other risk factors, and early response to treatment were analyzed with χ^2^ test and Fisher’s exact test. The main endpoints were event-free survival (EFS) and relapse-free survival (RFS), and overall survival (OS). EFS was defined as the time from the date of diagnosis to the first event (disease progression, relapse, death). RFS was calculated from the date of complete remission to the date of relapse. OS was defined as the time from the date of diagnosis to death. If no event occurred, the observation was censored at the last follow-up. The date of last follow-up was 31 December 2021. The Kaplan–Meier method was used to estimate survival probabilities; differences between groups were compared by log-rank test. The area under ROC curves was used to compare the usefulness of the time point MRD assessment in predicting relapse. The significance level of 0.05 was used in all statistical tests. Statistical analysis was performed using SPSS (Statistical Package for Social Science) version 27.0.

## 3. Results

### 3.1. Patients’ Characteristics

Table 3 presents an overview of the clinical and laboratory characteristics of All patients enrolled in the study. One hundred forty children, 53 girls, and 87 boys were finally analyzed. The median age at the time of diagnosis was 5.1 years (range 1.1–17.4). There were five children with Down syndrome (3.5%) in the study cohort. Most of the children were diagnosed with BCP-ALL 130 (93%) and 10 with T-ALL (7%). Among 140 children, 13 children were finally assigned to the SR group (9%), 98 to the IR group (70%) and 29 to the HR group (21%). The ETV6-RUNX1 mutation was detected in 34 children (24%), and hyperdiploidy in 30 children (21%). The rearrangement of KMT2A was detected only in four patients. In almost 14% of children detected genetic abnormalities were classified neither good nor poor risk factors. Most of the patients (23.5%) presented a normal karyotype of leukemic cells.

In all study patients, LAIP was successfully determined at diagnosis, thus further MFC MRD monitoring was feasible. Samples for analysis were available from all patients (n = 140) on day 15 (TP1), 139 (99%) children on day 33 (TP2), and 120 (86%) children on day 78 (TP3).

### 3.2. Outcomes

The median follow-up was 179 (range 22–279) months. The median follow-up for those patients who didn’t die or relapse was 186.5 (90.5–279). All patients achieved CR. There were 25 relapses (18%) in patients, mostly isolated (24 out of 25; twenty-one in the BM, three in the CNS, one in the testes). No deaths during induction or further stages of treatment were reported. Eleven patients died, all after relapse; six due to disease progression, one due to GVHD after HSCT and four due to fatal sepsis (including two patients with Down syndrome). There were two secondary malignancies among study patients: acute myeloid leukemia and disseminated juvenile xanthogranuloma and hemophagocytic lymphohistiocytosis. Table 4 presents an overview of events and 5-year outcome data from study patients.

#### 3.2.1. Outcome by Patient Characteristics

In our study, boys had a worse outcome, although the differences were not statistically significant (EFS 82.8% vs. 89.9%, *p*-0.32; and OS 94.4% vs. 98.1%, *p*-0.42, respectively). There were no significant differences in treatment outcomes either in the respect of the CNS initial infiltration (CNS3 status), poor response to prednisone, nor age above 15. Significantly inferior EFS and RFS had patients with the T-ALL immunophenotype compared to BCP-ALL patients (EFS, 50.7% vs. 88.0%, *p* < 0.05, and RFS, 49.2% vs. 85.6%, *p* < 0.05, respectively). In our study, the high initial leukemic burden (≥50,000 leukemic cells/μL) was a poor prognostic factor (significantly lower OS 86.9% vs. 97.5%, respectively; *p* < 0.05).

#### 3.2.2. Outcome by Risk Group

In the SR group (n = 13), the least numerous, no deaths nor relapses were observed. The treatment outcome by risk groups was shown in Table 4 and Figures S2 and S3 in Supplementary Materials.

#### 3.2.3. Outcome by MRD Status

On day 15, MRD was below 0.01% in 7% of children, between 0.01 and 0.1% in 11% of patients, between 0.1 and 1% in 28% and more than 1% in 54% of patients. The MRD on day 33 was below 0.01% in 68% of the children, between 0.01 and 0.1% in 21.5% of the patients, between 0.1 and 1% in 8.5%, and more than 1% in 2% of the patients. (Table 5) Among the patients who had MRD (>0.01%) on day 33, 22.2% (10 children) had favorable cytogenetics, 33.3% (15 children) had high-risk cytogenetics, and 44.4% (20 children) had no genetic abnormalities or changes of unknown prognostic impact. Two patients with positive MRD on day 78 presented unfavorable cytogenetics [one patient KMT2A, the other hypodiploidy]. Most patients with residual disease below 0.1% on day 15 had hyperdiploidy (16 out of 26) or ETV6-RUNX1 (10 out of 26) in ALL cells. Twenty-eight of 30 children with hyperdiploidy and 30 of 34 with ETV6-RUNX1 presented MRD below 0.1% at EOI. The presence or absence of MRD on day 33 was significantly related to the white blood cell count at the time of diagnosis (*p* = 0.02), the risk group (*p* = 0.02), while MRD on day 15 with the risk groups (*p* < 0.01), blast cells on day 8 (*p* = 0.003). White blood cell counts ≥ 50,000/μL, high risk group, and poor prednisone response status were significantly corelated with MRD above 0.1%. (Table S1, Supplementary Materials).

**Figure 1:**
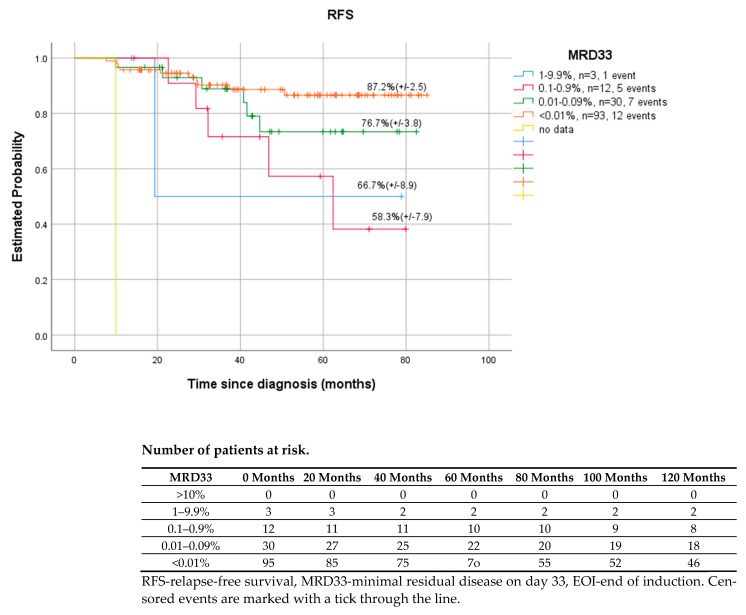
Relapse free survival of patients with respect to MRD33 (EOI), *p* < 0.01.

**Figure 2:**
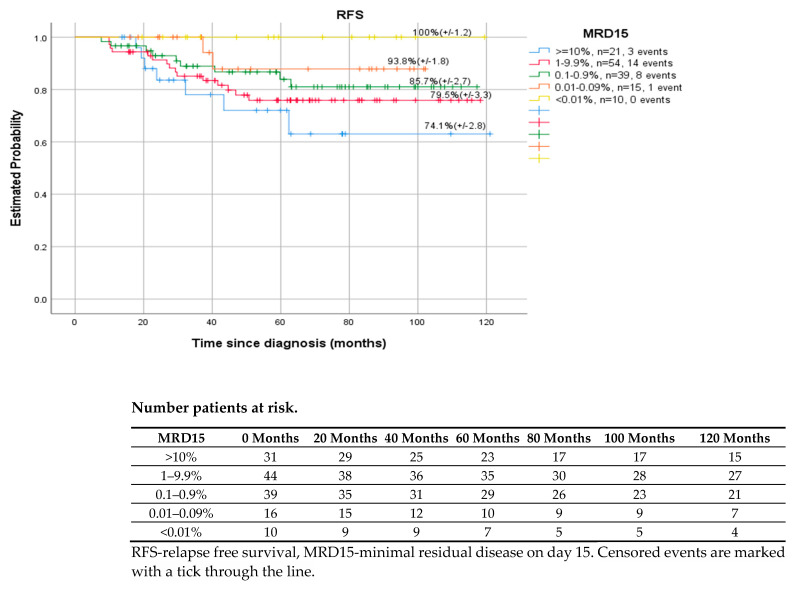
Relapse free survival of patients with respect to MRD15, *p* = 0.269.

Patients with no residual disease cells detected at EOI (day 33) had significantly higher 5-year RFS than MRD positive patients (>0.01%), (87.2% vs. 66.7%, *p* < 0.001). However, there were differences between specific ranges of MRD values. Children with MRD between 1–9.9% cells had better outcomes—RFS, EFS, compared to those with MRD level one log lower −0.1–0.9% (66.7% vs. 58.3%, *p* < 0.001). (Figure 1) The possible explanation for this could be the fact that most of the (8/12) patients with MRD 1–9.9% in EOI were finally classified as HRG and received more extensive post-consolidation treatment (HR blocks and 3 out of 12 also HSCT), which improved the outcome of that group. Similar differences in results were observed on day 15 and day 78 of MRD. (Figure 2 shows the results data referring to MRD on day15).

**Figure 3:**
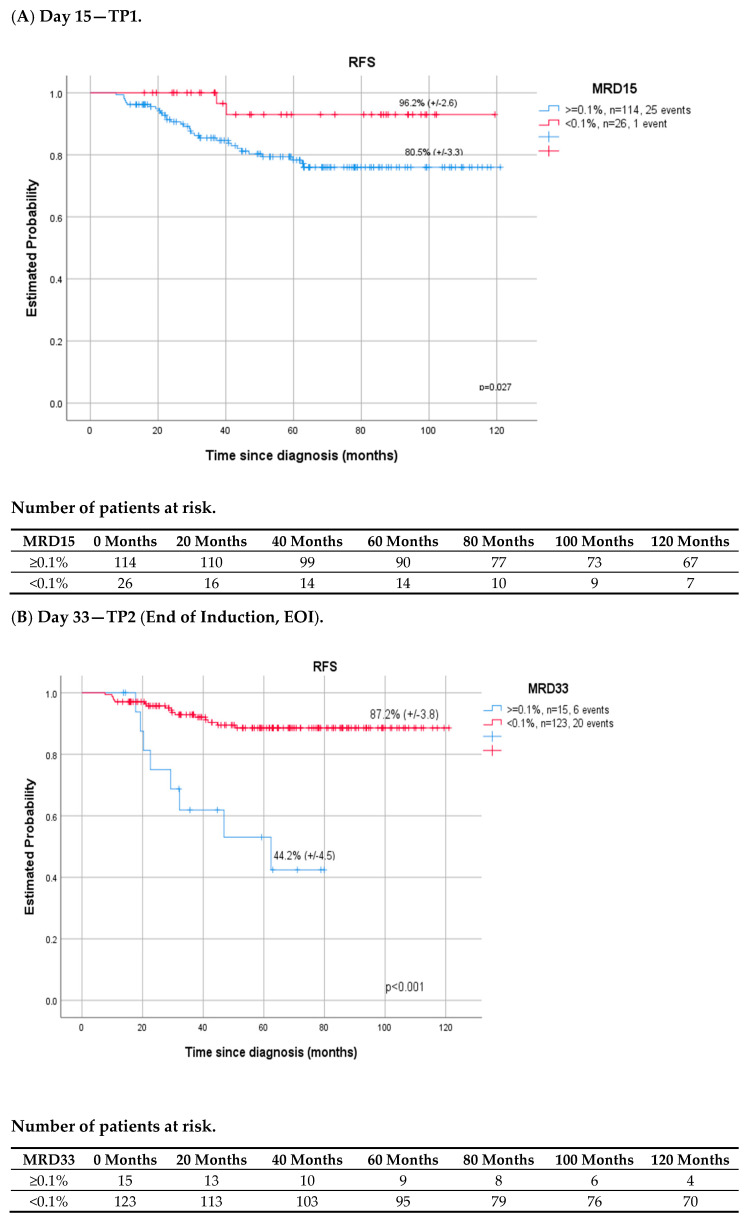

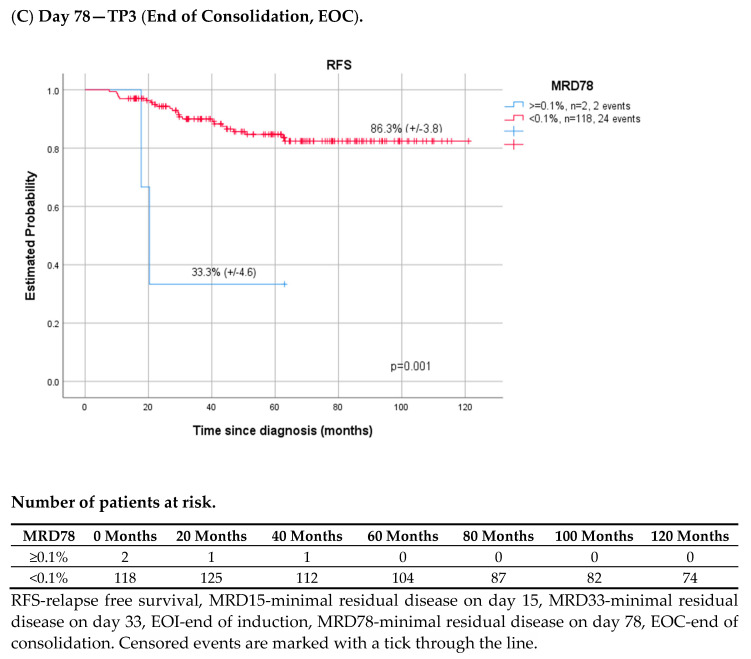
Relapse-free survival (n-140), according to MRD response (≥0.1% vs. <0.1%) on day (**A**) day 15 (TP1), (**B**) day 33 (TP2), (**C**) day 78 (TP3).

In our study, the 0.1% MRD cut-off was the most discriminatory for all patients at all time points evaluated. On day 15 patients with MRD > 0.1% have a lower OS and RFS rate compared to patients with MRD < 0.1% (93.1% vs. 100%, *p* = 0.08; 78.2% vs. 96.2%, *p* = 0.027, respectively). At EOI and EOC, the difference in OS and RFS rates between children with MRD below 0.1% and those with MRD < 0.1% was even clearer and reached statistical significance (Table 6, Figure 3A–C).

**Figure 4:**
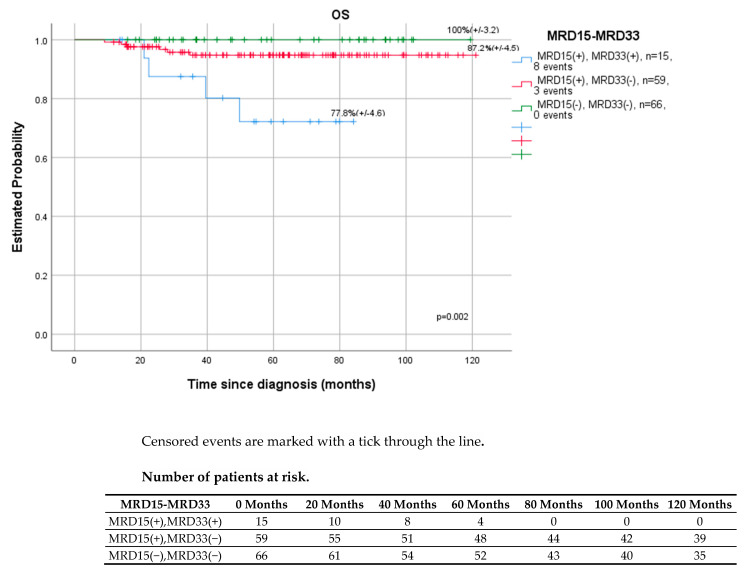
Outcomes of patients (n-140), 5y OS, by response to MRD (negative, <0.01% vs. positive, ≥0.01%) on days 15 (TP1) and 33 (TP2).

**Figure 5:**
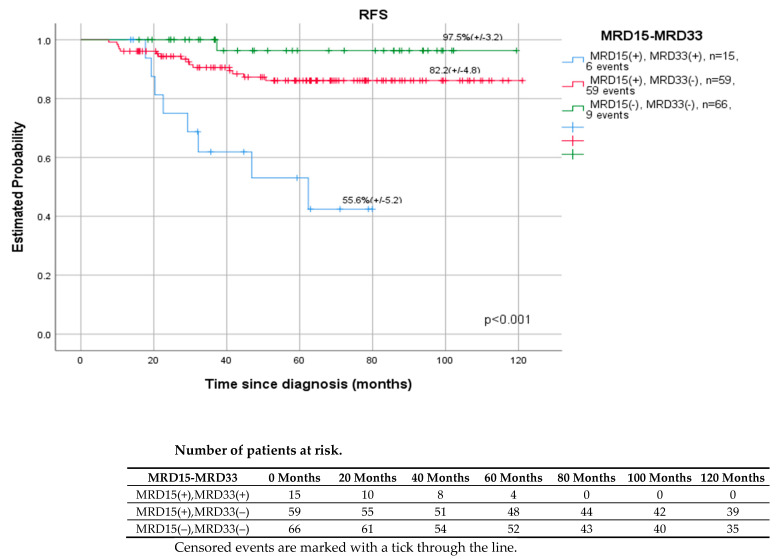
Outcomes of the patients (n-140), 5y RFS, by response to MRD (negative, <0.01% vs. positive, ≥0.01%) on days 15 (TP1) and 33 (TP2).

Figure 4 and Figure 5 present the results by the kinetics of the early blast clearance between day 15 and day 33/EOI. Patients with negative MRD on days 15 and 33 had a higher 5-year OS rate of 100% (*p* = 0.002) and RFS rate (97.6%, *p* < 0.001) than those with positive (≥0.01%) MRD at both time points (OS-77.8%, RFS-55.6%).

**Figure 6:**
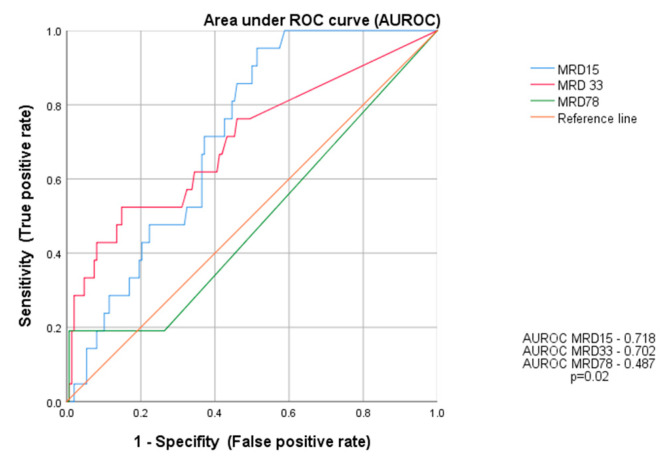
The area under the ROC curve (AUROC) for MRD assessment at days: 15 (TP1), 33 (TP2), and 78 (TP3).

In the analysis of prognostic significance of MRD assessment, days 15 and 33 compared to day 78 were more informative in predicting relapse. The area under the ROC curves (Figure 6) was 0.718; 0.702; and 0.487; respectively, indicating day 15 as the most informative in terms of the risk assessment of treatment failure in our group of patients, reflecting the probability of relapse, the differences were statistically significant (*p* = 0.02).

## 4. Discussion

Monitoring of MRD during induction and consolidation of acute lymphoblastic leukemia is a standard of care in many modern therapeutic protocols, both in children and adults. The evaluation of end-of-induction MRD is the single strongest prognostic factor [8,9,12,30]. The detection of immunoglobulin and T cell receptor gene rearrangements by quantitative real-time PCR (RT-PCR) is considered the gold standard for MRD evaluation [4,6,16,17,18,19,23]. However, MFC method, which is more accessible, also shows satisfactory performance. In contrast to PCR methods, MRD FMC is applicable to virtually all patients, which was also shown in our study. MRD assessment was feasible in all children and LAIP was successfully determined at diagnosis, allowing further analysis. False negative results due to immunophenotypic changes were prevented by using a wide combination of important number of LAIP antigens per patient. The downmodulation reported by other study groups of CD10, CD34, CD19 and upmodulation of CD45, CD11a, CD20 were also observed in our study, although accurate residual burden assessment was still feasible [31,32,33]. Regardless of the method used, MFC, PCR, or NGS, the assessment of MRD was the most reliable tool in pediatric ALL to identify patients for whom HSCT is indicated in the first CR and those who can be cured with standard chemotherapy alone [12,14,15,16,17,18,34]. The precise time points at which MRD is measured and the threshold used for treatment decisions differ between therapy protocols and the MRD detection method [8,9,13,15,16,17,18,19,20]. Our research confirmed the significance of MFC MRD on day 33 in the bone marrow as the most powerful early predictor of relapse (Figure 1). However, in the ROC curve analysis, which helped to find the most accurate assessment time point characterized by the highest sensitivity to predict relapse (Figure 6), day 15 and 33 were shown to be comparably significant (AUROC 15 vs. AUROC 33, 0.718 vs. 0.702). As shown per Figure 4 and Figure 5 both MRD on both days: 15 and 33 were predictive of overall survival and relapse. In children with negative MRD at both time points, day 15 and 33 (TP1 and TP2) had higher OS and RFS rates compared to those with positive MRD levels (≥0.01%) at both time points (OS—100% vs. 77.8%, *p* = 0.002; RFS—97.6% vs. 55.6%, *p* < 0.001).

In the AEIOP-BFM–ALL study by Basso and colleagues, the prognostic significance of day 15 was shown [13]. Despite the wide implementation of the PCR assessment of MRD at days 33 and 78 (end of induction and consolidation) in contemporary BFM-based chemotherapy for ALL, day 15 MFC MRD is still an important tool that highlights the prognostic importance of early response to therapy [14,16,29]. In our study, MRD measured by MFC in bone marrow on day 15 was used additionally for risk assignment. The number of patients with SR treated according to ALL IC-BFM 2002 protocol, the former protocol used before ALL IC-BFM 2009, decreased by almost half compared to the number of patients with SR treated according to the ALL IC-BFM 2009 protocol. Of 98 IRG patients 39 would have been stratified to SRG according to former criteria of the risk groups, whereas MRD status > 0.1% on day 15 finally allocated those patients to IRG. All 13 patients of SRG would have been allocated to standard group anyway, based on age, initial WBC, response to prednisone and BM morphology on day 15 and 33. Concluding, the stratification based on the assessment on day 15, MRD < 0.1%, allowed for a better separation of patients with the best prognosis, although in our study cohort none patients would have been “shifted” from IRG to SRG based on MRD < 0.1%, on day 15, which means sparing two doses of daunorubicin in induction.

Results of the SRG patients in our study were outstanding, 5-year OS and EFS were both 100%, and the MRD threshold below 0.1% on day 15 allowed, in fact, to find children with very low risk of relapse and reduce induction treatment (spared two doses of daunorubicin). The reduced intensity approach was used in many studies [14,15,16]. Among others, Pedrosa et al. showed that a very low risk of relapsed children identified with simplified flow cytometry, with MRD on day 19 of <0.01% possibly benefiting from a mildly myelosuppressive chemotherapy regimen [27].

Postinduction MRD status was shown to be a powerful independent prognostic factor in all subtypes of ALL, and many studies showed MRD superiority over well-known, historically relevant prognostic factors, such as age, initial white blood cell count, and cytogenetics [8,9,17]. This was also confirmed by our study, as patients with MRD less than 0.01% on day 33 (EOI) showed a significantly better outcomes compared to children with residual cell ≥ 0.01%, (87.2 vs. 66.7%, respectively, *p* < 0.001). In our cohort, the 0.1% MRD threshold was the most discriminatory at all assessed time points. Basso et al., in the AEIOP-BFM2000 study, showed that approximately 40% of patients with childhood ALL treated with contemporary BFM-based chemotherapy achieve subtotal clearance of leukemic cells (<0.1% among bone marrow nucleated cells) after 14 days of therapy (on day 15), and have excellent treatment outcomes, with a cumulative 5-year incidence of relapse of 5.4% [13]. The Children’s Oncology Group reported a large study of MRD-MFC on peripheral blood day 8, the end of induction (29 day), and the end of bone marrow consolidation, in children with BCP-ALL. The MRD (≥0.1%) on day 8 and day 29 bone marrow was associated with poor EFS in all patients [12].

When interpreting our results, one must know about some limitations of our study. This is a single-center study with a relatively small group of patients, presenting treatment outcomes according to the ALL IC-BFM2009 protocol. An important limitation of the current study was also the incomplete genetic testing in some patients. The study was carried out over time, when genetic assay was not available for some patients, and some genetic abnormalities were not tested, such as IKZF1 mutations. That could a be also probable cause of treatment failure in some children with negative MRD.

Pui et al. showed that the clinical impact of MRD on the outcome varies according to the immunophenotype and genotype of leukemia, as well as the time of the measurements, so a single result of MRD should not be taken as a decisive prognostic factor. They reported that patients with ETV6-RUNX1 and those with hyperdiploidy and negative MRD on day 19 (after two weeks if induction) had a particularly low risk of relapse: 1.9% and 3.8%, respectively. Children with high-risk B-cell ALL (BCR-ABL1 positive, age ≥ 10 years or a leukocyte count ≥ 50 × 10^9/^L) and T-cell ALL, even with negative MRD on day 46 (EOI) had an inferior outcome (cumulative risk of relapse 12.7% and 15.5%, respectively. However, 16% of children with hyperdiploid ALL and MRD ≥ 1% at day 19 had a high risk of relapse (23.5%) [20]. Xue et al. also showed that patients with hypodiploid ALL and MRD ≥ 10% at day 15 had poorer EFS (63.6%) and OS (65.9%) than those with lower MRD. In particular, children with ETV6/RUNX1, even those with slow disease clearance, had a particularly low cumulative incidence of relapse, 8.3% [35]. In our study, patients with low-risk genetics (ETV6-RUNX1 or hyperdiploidy) presented early clearance of the disease (absence of MRD on day 15 and 33), while high-risk genetics, KMT2A, and hypodiploidy were detected in patients with positive MRD on day 78, EOC. A similar dependency between disease clearance and genetics of leukemic cells was observed by O’Conor et al. Children with good-risk genetics, especially those with ETV6-RUNX1, had the fastest clearance, whereas patients with high-risk genetics (KMT2A fusions, or hypodiploidy) showed the slowest clearance of leukemic cells [3]. In the study by Jeha et al., 5-year OS for patients with ETV6-RUNX1 or high-hyperdiploid ALL exceed 99% [99.2% and 99.4%, respectively] [36].

Notably, there were 25 relapses in our study, and almost half of them (10/25) occurred among patients who were negative for MRD on day TP2 and TP3. Three of those relapses were isolated CSN and one in the testes, reflecting the location in isolated chemoresistant sanctuaries, not detectable with MRD techniques based on cells from bone marrow or peripheral blood. Although MRD is a direct measure of the burden of the disease and the response to treatment in ALL, there may be sanctuary sites (in the brain or testes) that also contribute to relapse and may not be measurable by marrow or blood MRD. In our study there were isolated relapses (testes, CSN) among patients with negative MRD at all measured points on day 15, 33, and 78).

## 5. Conclusions

Whitlock used the term “Go with the flow” for the first time, who advised this method as a reasonable option for the assessment of MRD for low- and middle-income countries. Simplified MFC MRD and limited genetic analysis-directed therapies allow one to introduce MRD-directed therapies and identification of children of very low risk of relapse [28]. The BFM group showed that a single marker PCR MRD analysis may be adequate for the evaluation of prognoses in ALL patients, which was also confirmed by others, for example, in the Malaysia–Singapore ALL 2003 study [24]. However, this approach could still be beyond the capabilities and resources of many developing countries, thus a simplified flow cytometric method could be used along with genetic analysis for MRD-directed therapies. Democratization of access makes MFC MRD easily accessible, a rapid method of assessment of live cells feasible in many centers, making the term “go with the flow” possible to implement in daily clinical practice.

In conclusion, MRD-selected treatment improves outcomes in children and adults with ALL. The rest of leukemic cells resistant to chemotherapy could initiate relapse. The measurable disease reflects the response to administered therapy and inform the need to intensify or de-intensify the treatment. We showed the assessment of MFC MRD on day 33, the strongest prognostic factor for children treated according to the ALL IC-BFM2009 protocol, together with the clearance of leukemic blast between days 15 and 33 of induction treatment. Patients with low-risk genetics (ETV6-RUNX1 or hyperdiploidy) presented early clearance of the disease (absence of MRD on days 15 and 33), while high-risk genetics, KMT2A, and hypoploidy were detected in children with positive MRD at EOC. The precise time points of MRD measurements and the predictive value of MRD should always be carefully determined in the context of each treatment regimen, as well as the genotype and immunophenotype of leukemia.

## Figures and Tables

**Table 1:** A, B, C. Panels of monoclonal antibodies used for MRD monitoring.

**Panel A.** For BCP-ALL, six-color tubes:
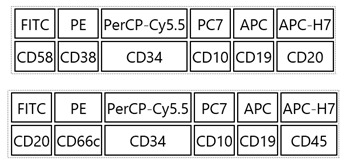
**Panel B.** For BCP-ALL, ten-color tubes:
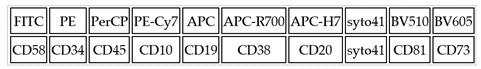
**Panel C.** For T-ALL, six-color tubes:
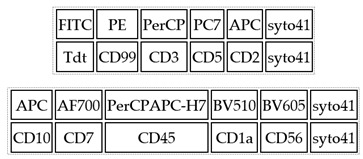

**Table 2:** Risk group assignment according to ALL IC-BFM2009 protocol.

Risk Group	Criteria
**Standard risk group (SRG) ^1^**	PB day 8: <1000 blasts/µL age ≥1 yr—6 yr initial WBC < 20,000/µL if available, MFC MRD < 0.1% or bone marrow M1/M2 on day 15 and no bone marrow of M2/M3 on day 33
**Intermediate risk group (IRG)** **High risk group (HRG) ^2^**	All patients who are not stratified for SR or HR are intermediate-risk patients. IR and, if available, MFC MRD > 10% or M3 bone marrow on day 15 SR if MFC MRD > 10% PB on day 8: ≥1000 blasts/µL M2 or M3 bone marrow on day 33 t(4;11) [KMT2A-AFF1] Hypodiploidy ≤ 44

^1^ All criteria must be fulfilled. ^2^ At least one criterion must be fulfilled. SRG—standard risk group, IRG—intermediate risk group, HRG—high risk group, PB—peripheral blood, WBC—white blood cells, MFC MRD—minimal residual disease measured by multiparametric flow cytometry, M1—bone marrow containing less than 5% blasts, M2—bone marrow containing 5–25% blasts, M3—bone marrow containing more than 25%.

**Table 3:** Clinical and laboratory characteristics of study patients.

Features	Protocol ALL IC-BFM2009 n = 140
**Age in years**	
Median (range)	5.1 (1.1–17.4)
**Sex [n (%)]**	
Boy	87 (62%)
Girls	53 (38%)
**Down syndrome [n (%)]**	5 (3.5%)
**WBC count (×10^9^/L)**	
Median (range)	10.2 (1.0–776)
**Immunophenotype [n (%)]**	
BCP-ALL	130 (93%)
T-ALL	10 (7%)
**CNS [n (%)]**	
CNS 1	130 (93%)
CNS 2	5 (3.5%)
CNS 3	5 (3.5%)
**Risk group [n (%)]**	
Standard risk (SR)	13 (9%)
Intermediate risk (IR)	98 (70%)
High risk (HR)	29 (21%)
**Genotype [n (%)]**	
Negative *	19 (14%)
ETV6-RUNX1	34 (24%)
KMT2A	4 (3%)
TCF3-PBX1	2 (1%)
PAX5-ETV6	1 (<1%)
Hyperdiploidy (>50 chromosomes)	30 (21%)
Hypodiploidy (<44 chromosomes)	1 (<1%)
Normal karyotype	33 (23.5%)
No data	16 (11.5%)
**Prednisone response [n (%)]**	
PGR	121 (86%)
PPR	19 (14%)
**Complete remission [n (%)]**	140 (100%)
**Death [n (%)]**	11 (8%)
**Relapse [n (%)]**	25 (18%)
**BM relapse**	20
**CNS relapse**	3
**Relapse of testes**	1
**BM + CNS relapse**	1
**Secondary malignancies**	2

BM—bone marrow, CNS—central nervous system, PGR—prednisone good response, PPR—prednisone poor response, BCP-ALL—ALL B cell precursor, negative genotype *—presence of genetic abnormalities of not known impact on prognosis.

**Table 4:** Outcome data by patient characteristics for children treated according to ALL IC-BFM 2009 (n-140).

Features	OS 5-Year % (SE)	*p*	RFS 5-Year % (SE)	*p*	EFS 5-Year % (SE)	*p*
**Sex**						
Boy	94.4 (2.1)	0.42	81.8 (3.4)	0.32	89.9 (3.5)	0.32
Girls	98.1 (1.8)		87.1 (3.5)		82.8 (3.3)	
**Immunophenotype**						
BCP-ALL	96.6 (1.5)	0.39	85.6 (2.4)	**0.003**	88.0 (2.4)	**0.005**
T-ALL	70.0 (4.7)		49.2 (9.8)		50.7 (9.6)	
**CNS**						
CNS 1 + 2	96.6 (1.5)		84.9 (2.5)		87.3 (2.5)	
CNS 3	79.2 (12.9)	0.1	63.7 (16.1)	0.16	65.5 (15.7)	0.17
**Risk group**						
Standard risk (SR)	100		100 (2.5)		100 (2.5)	
Intermediate risk (IR)	92.9	0.5	79.6 (3.5)	0.22	79.6 (3.5)	0.21
High risk (HR)	96.6		79.3 (7.4)		79.3 (7.4)	
**Prednisone response**						
PGR	96.2 (1.6)	0.95	84.2 (2.7)	0.7	86.7 (2.7)	0.7
PPR	91.2 (3.3)		77.1 (6.8)		79.1 (6.9)	
**Age**						
<15 yr	95.9 (1.5)	0.41	84.1 (2.6)	0.69	85.5 (2.5)	0.69
≥15 yr	91.6 (8.7)		81.3 (10.4)		84.2 (10.5)	
**WBC at diagnosis**						
<50,000/μL	97.5 (1.4)	0.04	87.0 (2.4)	**0.001**	89.4 (2.4)	**0.002**
≥50,000/μL	86.9 (6.0)		64.0 (8.9)		65.7 (8.9)	

Bolded means statistically significant.

**Table 5:** MRD levels in bone marrow on day 15, day 33, and day 78.

MRD	Day 15	Day 33 (EOI)	Day 78 (EOC)
≥10% (10^−1^)	31 (22%)	0	0
1–10% (10^−2^–10^−1^)	44 (31.5%)	3 (2%)	0
0.1–1% (10^−3^–10^−2^)	39 (28%)	12 (8.5%)	0
0.01–0.1% (10^−4^–10^−3^)	16 (11.5%)	30 (21.5%)	2 (1.5%)
≤0.01% (<10^−4^)	10 (7%)	95 (68%)	136 (98.5%)

EOI—end of induction, EOC—end of consolidation.

**Table 6:** Outcomes by MRD result: MRD ≥ 0.1% vs. MRD < 0.1% on day 15 (TP1), 33 (TP2) and 78 (TP3).

Day 15 (TP1)			Day 33 (TP2)		Day 78 (TP3)	
	MRD ≥0.1%	MRD < 0.1%	MRD ≥0.1%	MRD <0.1%	MRD ≥0.1%	MRD < 0.1%
**5yOS**	93.1	100	86.7	95.2	33.3	95.2
	*p* = 0.08		***p* = 0.027**		***p* = 0.001**	
**5yRFS**	80.5	96.2	44.2	87.2	33.3	86.3
	***p* = 0.027**		***p* = 0.008**		***p* = 0.001**	
**5yEFS**	80.9	96.2	44.2	87.2	33.3	86.3
	*p* = 0.097		***p* = 0.009**		***p* = 0.001**	

Bolded means statistically significant.

## Data Availability

The data are not publicly available due to privacy and ethical restrictions.

## Supplementary Materials

The following supporting information can be downloaded at: https://www.mdpi.com/article/10.3390/cancers14215359/s1, Figure S1: Gating strategy used in monitoring of MRD in children with acute lymphoblastic leukemia treated according to ALL IC-BFM2009; Figure S2: Overall survival of patients with respect to risk groups (n-140), *p* = 0.5; Figure S3: Event free survival of patients with respect to risk groups (n-140), *p* = 0.215; Table S1: MRD status on day 15, day 33 (End of induction) and day 78 (End of consolidation) by age, initial WBC, risk groups, BM morphology on day 15 and 33;
