# Supplementary material for: Go with the Flow—Early Assessment of Measurable Residual Disease in Children with Acute Lymphoblastic Leukemia Treated According to ALL IC-BFM2009
Source: Cancers (Basel). 2022 Oct 30;14(21):5359. doi: 10.3390/cancers14215359 (PMC9653819; doi:10.3390/cancers14215359)
Supplement: Supplementary file 1 [file cancers-14-05359-s001.zip › cancers-1898127-supplementary.pdf]

Supplementary materials

Figure S1. Gating strategy used in monitoring of MRD in children with acute lymphoblastic leukemia treated according to ALL IC-BFM2009

Panel a) Normal bone marrow - B cells are gated based on CD19 expression and healthy B cell progenitors are gated based on CD45 and CD10 expression. This population is then shown on the following plots to establish the pattern of expression of all the other antigens on the normal hematogones.

Panel b) and c) The patient samples are analyzed using the same set of gates as established on the normal control, then patient-specific antigen expression patterns that best discriminates patient leukemia blasts from normal hematogones are established and additional gates are set. Hence, there are two sets of gates per patient in the dotplots - the same as for the healthy control and the patient-specific ones. Finally, gates that most accurately distinguish the patient's pathological cells from normal cells are merged and calculated as percentage of nucleated cells.
In case of immunophenotypic shifts resulted from treatment (e.g, decreased of CD10 or CD19 expression), gates are adjusted, or gating is changed in accordance to newly formed phenotype, but still searching "different from normal" events. Panel a) Normal bone marrow - B cells are gated based on CD19 expression and healthy B cell progenitors are gated based on CD45 and CD10 expression. This population is then shown on the following plots to establish the pattern of expression of all the other antigens on the normal hematogones.

A. **Panel A - Normal bone marrow**

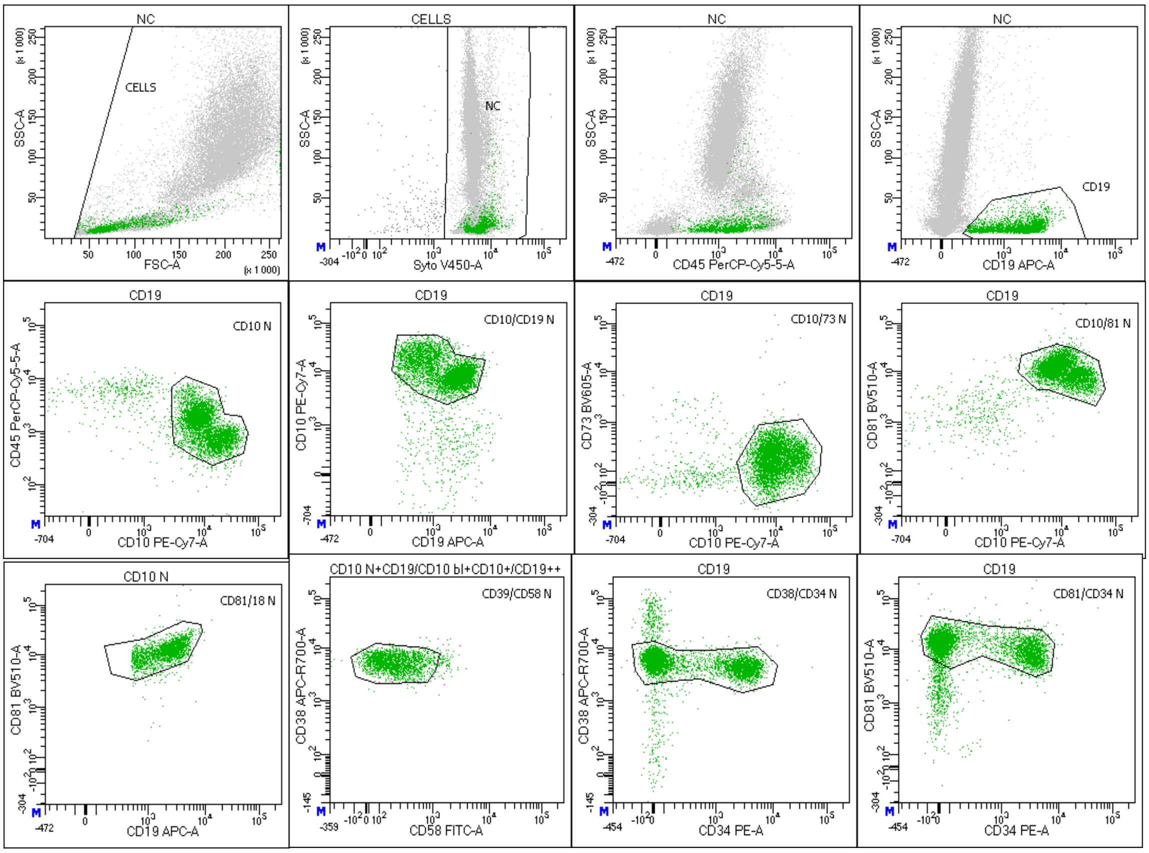

B. Panel B for gating BCP-ALL

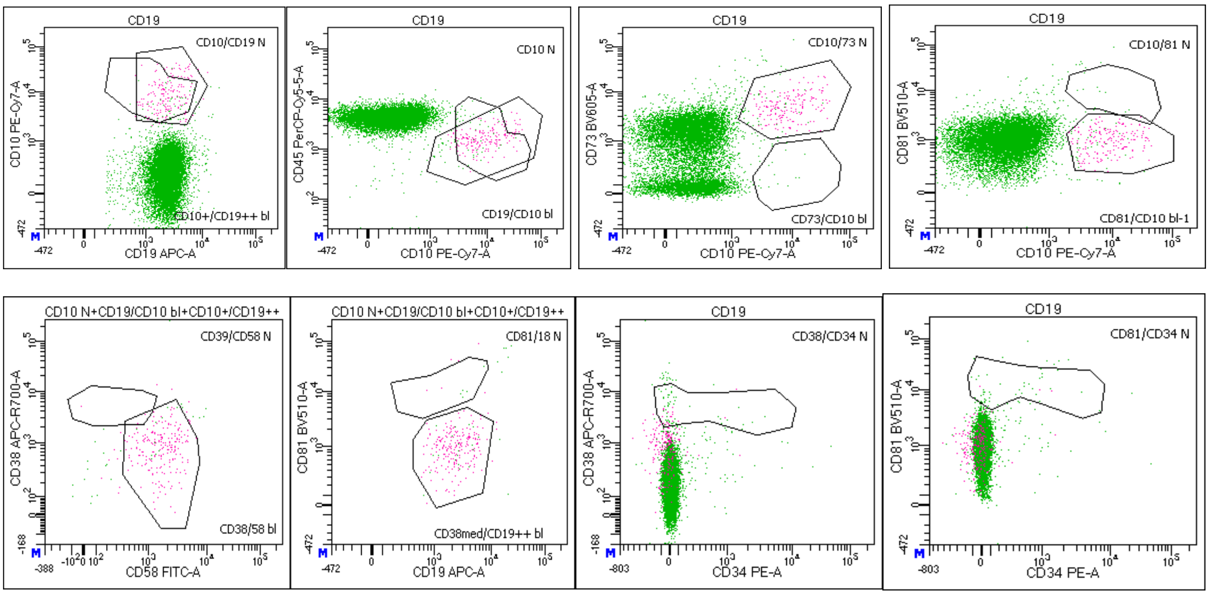

C. Panel C for gating BCP-ALL

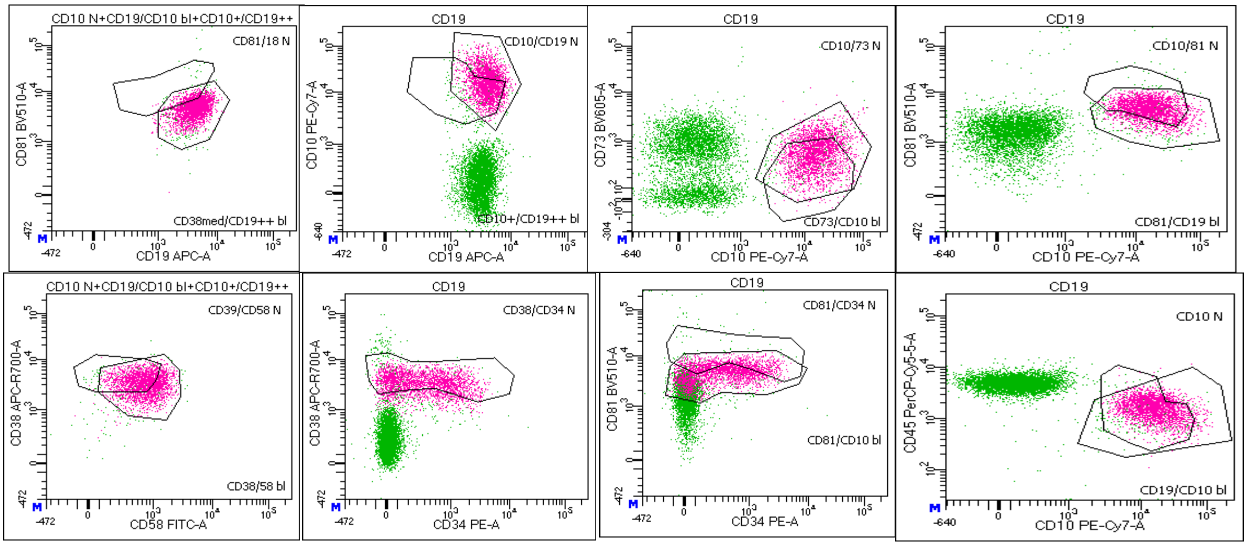

BCP – B-cell precursor leukemia

Figure S2. Overall survival of patients with respect to risk groups (n-140), p=0.5.

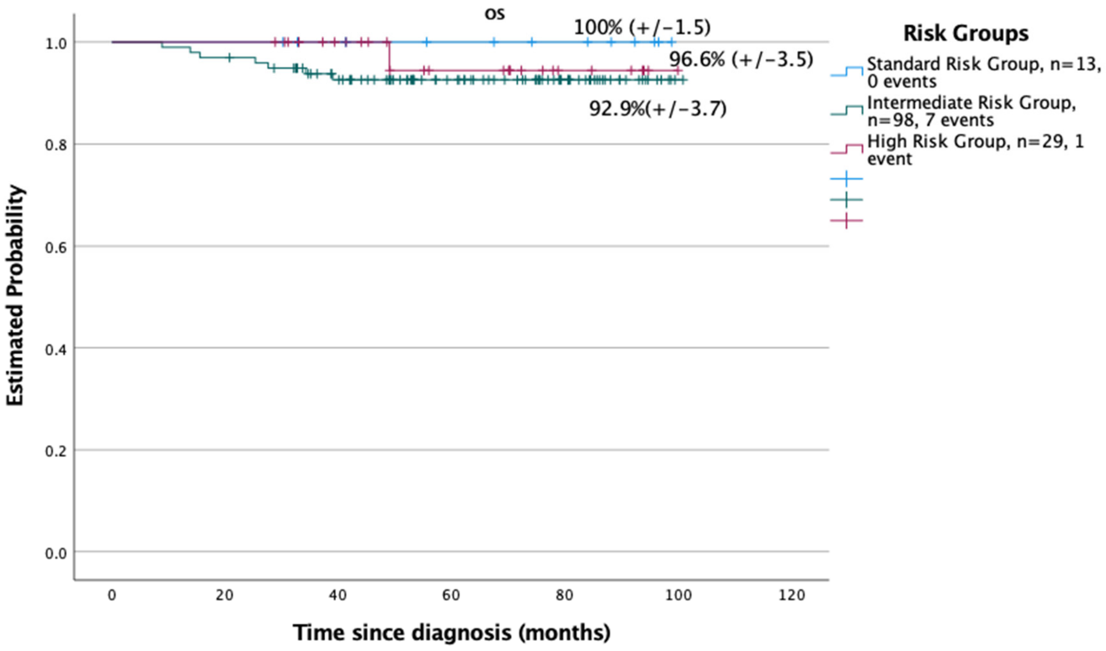

OS – overall survival. Censored events are marked with a tick through the line.

Number of patients at risk.

Risk groups	0 months	20 months	40 months	60 months	80 months	100 months	120 months
SRG	13	13	12	11	10	10	9
IRG	98	93	51	48	43	40	38
HRG	29	20	50	45	34	32	27

Figure S3. Event free survival of patients with respect to risk groups (n-140), p=0.215.

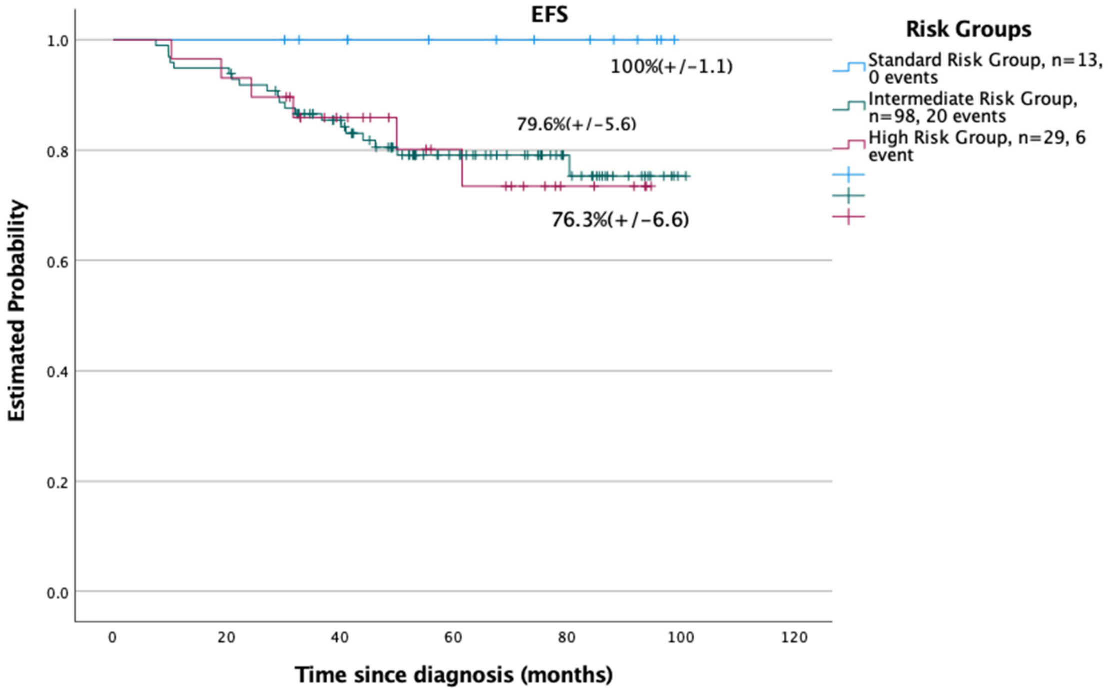

EFS – event free survival. Censored events are marked with a tick through the line.

Number pf patients at risk.

Risk groups	0 months	20 months	40 months	60 months	80 months	100 months	120 months
SRG	13	13	12	11	10	10	9
IRG	98	93	51	48	43	40	38
HRG	29	20	50	45	34	32	27

Table S1. MRD status on day 15, day 33 (End of induction) and day 78 (End of consolidation) by age, initial WBC, risk groups, BM morphology on day 15 and 33.

	MRD15 (n-140)		*p*	MRD33 – EOI (n-139)		*p*	MRD78 -EOC (n-120)		*P*
	≥0.1%	<0.1%		≥0.1%	<0.1%		≥0.1%	<0.1%	
**Age at diagnosis**									
<6 y	64/82	18/82	*0.198*	9/81	73/81	*0.546*	0	69/69	*0.66*
≥6 y	45/58	13/58		7/58	51/58		2/58	48/50	
WBC									
< 50 000/µl	96/120	24/120	*0.321*	10/119	109/119	***0.02***	1	101/102	*0.473*
≥50 000 /µl	16/20	4/20		12/20	4/20		1	17/18	
**Risk Groups**									
SRG	1/13	12/13	***<0.01***	0	13/13	***0.02***	0	13/13	*0.89*
IRG	82/98	16/98		91/97	6/97		0	85/85	
HRG	26/29	3/29		10/29	19/29		2	21/23	
**Prednisone response**									
PGR	98/121	23/121	***<0.01***	12/108	108/120	*0.089*	1	105/106	*0.761*

PPR	18/19	1/19		10/19	9/19		1	13/14	
**Blasts in bone marrow on day 15**									
M1	78/104	26/104	*0.061*	6/103	97/103	*0.067*	0	99/99	*0.069*
M2	28/30	2/30		6/30	24/30		2	14/16	
M3	6/6	0		3/6	3/6		0	5/5	
**Blasts in bone marrow on day 33**									
M1	107/137	30/137	*0.081*	5/136	131/136	*0.088*	2	115/117	*0.066*
M2	½	1/2		0	2/2		0	2/2	
M3	1/1	0		1/1	0		0	1/1	
**Genetics**	n=69			n=69			n=69		
Favorable risk genetics	38/64	26/64	*0.451*	6/64	58/64	*0.342*	0	64/64	*0.358*
Poor risk genetics	5/5	0/5		3/5	2/5		2/5	3/5	

Favorable risk genetics – ETV6-RUNX1, hyperdiploidy; **Poor risk genetics** – KMT2A, hypodiploidy; **n -** number of samples analyzed on each time point, **WBC** - white blood count, **SRG** - standard risk group, **IRG** – intermediate risk group, **HRG** – high risk group, **PGR** - prednisone good response, less than 1000 blasts in 1 µl blood on day 8 of induction, **PPR** – more or equal 1000 blasts in 1 µl blood on day 8 of induction therapy, **M1** - bone marrow with less than 5% blasts, **M2** - bone marrow with 5-25% blasts, **M3 -** bone marrow with more than 25% blasts.
